# Proteolytic cleavage of p53 mutants in response to mismatched DNA

**DOI:** 10.1038/sj.bjc.6690679

**Published:** 1999-09

**Authors:** T Mee, A L Okorokov, S Metcalfe, J Milner

**Affiliations:** YCR P53 Research Group, Department of Biology, University of York, York, YO10 5DD, UK; Department of Surgery, University of Cambridge, Addenbrooke's Hospital, Cambridge, UK

**Keywords:** p53 mutants, proteolytic cleavage, mismatched DNA

## Abstract

Interaction of p53 with mismatched DNA induces proteolytic cleavage with release of a 35-kDa protein fragment from the p53–DNA complexes. The 35-kDa cleavage product is activated for specific biochemical function(s) and may play a role in the cellular response to DNA damage (Molinari et al (1996) *Oncogene***13**: 2077–2086; Okorokov et al (1997) *EMBO J***16**: 6008–6017). In the present study we have asked if mutants of p53 retain the ability to undergo similar proteolytic cleavage, and compared sequence-specific ‘DNA contact’ with ‘structural’ mutants commonly found in human cancer. In addition, a series of phosphorylation site mutants were generated to investigate the possible effects of phosphorylation/dephosphorylation on the proteolytic cleavage of p53. All mutants tested bound to a mismatched DNA target *in vitro*. Moreover, studies in vitro and in vivo indicate that p53 mutants with intact conformational structure (as determined by immunoreactivity with PAb246 and PAb1620) retain the ability to undergo proteolytic cleavage similar, if not identical, to the wild-type p53 protein. Our results suggest that the capacity for p53 to bind mismatched DNA is independent of structural conformation of the central core domain. Proteolytic cleavage, however, is crucially dependent upon a wild-type conformation of the protein. © 1999 Cancer Research Campaign


*British Journal of Cancer* (1999) **81**(2), 212–218
© 1999 Cancer Research Campaign
Article no. bjoc.1999.0679

### Proteolytic cleavage of p53 mutants in response to mismatched DNA

**T Mee[1], AL Okorokov[1], S Metcalfe[2] and J Milner[1]**

[1]YCR P53 Research Group, Department of Biology, University of York, York YO10 5DD, UK; [2]Department of Surgery, University of Cambridge, Addenbrooke's Hospital, Cambridge, UK

**Summary** Interaction of p53 with mismatched DNA induces proteolytic cleavage with release of a 35-kDa protein fragment from the p53–DNA complexes. The 35-kDa cleavage product is activated for specific biochemical function(s) and may play a role in the cellular response to DNA damage (Molinari et al (1996) *Oncogene* **13**: 2077–2086; Okorokov et al (1997) *EMBO J* **16**: 6008–6017). In the present study we have asked if mutants of p53 retain the ability to undergo similar proteolytic cleavage, and compared sequence-specific 'DNA contact' with 'structural' mutants commonly found in human cancer. In addition, a series of phosphorylation site mutants were generated to investigate the possible effects of phosphorylation/dephosphorylation on the proteolytic cleavage of p53. All mutants tested bound to a mismatched DNA target *in vitro*. Moreover, studies in vitro and in vivo indicate that p53 mutants with intact conformational structure (as determined by immunoreactivity with PAb246 and PAb1620) retain the ability to undergo proteolytic cleavage similar, if not identical, to the wild-type p53 protein. Our results suggest that the capacity for p53 to bind mismatched DNA is independent of structural conformation of the central core domain. Proteolytic cleavage, however, is crucially dependent upon a wild-type conformation of the protein.

© 1999 Cancer Research Campaign

**Keywords:** p53 mutants; proteolytic cleavage; mismatched DNA

The p53 protein is activated in response to DNA damage, triggering pathways leading either to cell cycle arrest or apoptosis (reviewed by Gottlieb and Oren, 1996; Ko and Prives, 1996; Levine, 1997). The central core domain of p53 (amino acids 102–292) can bind with sequence-specificity to DNA elements (Cho et al, 1994). In this way p53 can transactivate the expression of target genes which include *p21*, a cyclin-dependent kinase inhibitor (El-Diery et al, 1993), and *Bax*, a promoter of apoptosis (Miyashita and Reed, 1995). Additional evidence suggests that p53 can act as a tumour suppressor independently of its transactivation function (Caelles et al, 1994; Haupt et al, 1995; Haupt et al, 1996), though the mechanisms of this activity are not understood. In human cancer, mutant forms of p53 commonly lose the capacity for sequence-specific DNA binding which is required for the transactivation of target genes (Cho et al, 1994). These mutations affect the core domain and can be classified into two main groups: (i) DNA contact mutants which have an altered residue normally involved in making direct contact with the DNA target sequence, and (ii) structural mutants which have altered conformation of the central core domain (Cho et al, 1994). Conformational folding is crucial for the correct positioning of the DNA contact residues and structural perturbation of the core is predicted to disrupt the specific interaction with DNA target sequences, resulting in loss of transactivation function.

p53 can also bind sites of DNA damage, such as mismatches and double-strand DNA ends (Lee et al, 1995), as well as short single-stranded DNAs (Jayaraman and Prives, 1995). This binding involves the carboxy terminus of the p53 protein and interaction with mismatched DNA *in vitro* results in p53 undergoing a specific, limited proteolytic cleavage reaction (Molinari et al, 1996; Okorokov et al, 1997). The DNA bound fraction of the cleavage reaction contains full-length p53 and a 40-kDa fragment lacking the amino terminal domain of the protein. The supernatant fraction contains a 35-kDa fragment, which is released from the DNA/protein complex. This fragment lacks both the amino and carboxy terminal domains, and retains a conformationally wild-type central core domain. It has been proposed that the proteolytic cleavage may be autocatalytic and play a role in the activation of p53 in response to DNA damage in vivo since p53 fragments of similar size have been observed following treatment of cells with DNA damaging agents (Molinari et al, 1996; Okorokov et al, 1997).

In the present study, we have asked whether mutant p53 undergoes the same cleavage reaction as the wild-type protein when incubated with DNA in vitro. For this purpose we generated a series of murine p53 mutants by site directed mutagenesis. Each mutant was expressed in vitro using rabbit reticulocyte lysate for translation (according to Milner et al, 1991) and assayed for the capacity to bind mismatched DNA and undergo proteo[illegible] cleavage. The reason that murine, rather than human, p53 was chosen for this study is that the human protein produces a number of internal initiation products when translated in vitro, with a major product at 40 kDa. This complicates analysis of the proteolytic cleavage products induced in response to mismatched DNA. With murine p53 this problem is not encountered.

### MATERIALS AND METHODS

### Site-directed mutagenesis and subcloning

Mutagenesis was carried out using the vector pSELEC (Promega) with the murine *p53* gene cloned downstream of the T7

*Received 1 December 1998*
*Revised 16 March 1999*
*Accepted 17 March 1999*

*Correspondence to:* J Milner

**Table 1** DNA contact and structural mutants of murine p53

| Class of mutant | Murine mutant | Human equivalent | Mutagenesis oligonucleotide |
|---|---|---|---|
| DNA contact | R270H | R273H | 5′-ggacagctttgaggtgcacgtttgtgcctgccc-3′ |
| | R245H | R248H | 5′-cctgcatgggaggcatgaaccaccggccgatccttaccatcatcacactgg-3′ |
| | K117A | K120A | 5′-cttcctgcagtctgggaccgcggcgtctgttatgtgcacgtac-3′ |
| Structural | R172H | R175H | 5′-gacggaggtcgtgagacactgcccccaccatgag-3′ |
| | A135V | A138V | * |
| | C173F | C176F | * |
| | C173M | C176M | * |
| | C173D | C176D | * |

*Already available.

**Table 2** Phosphorylation mutants of murine p53

| Mutant | Kinase | Mutagenesis oligonucleotide | |
|---|---|---|---|
| N-terminus | S18A | DNAPK | 5′-gcctcgagctccctcttgcgcaggagacattttcaggc-3′ |
| | S18D | | 5′-gcctcgagctccctcttgatcaggagacatttcaggc-3′ |
| | S37D | JNK | 5′-ctccagaagatatcctgccagatcctcactgcatggacgatc-3′ |
| C-terminus | S312A | CDK | 5′-ctgcccacctgcacaagcgcagctcccccgcaaaagaaaaaacc-3′ |
| | S312D | | 5′-ctgcccacctgcacaagcgcagatcccccgcaaaagaaaaaacc-3′ |
| | S373A | PKC | 5′-gaagaccaagaagggccaggcgacgtcccgccataaaaaaacaatgg-3′ |
| | S373D | | 5′-ctgaagaccaagaagggccaggatactagtcgccataaaaaaacaatggtc-3′ |
| | S375A | | 5′-ccaagaagggccagtctaccgcgcgccataaaaaaacaatggtc-3′ |
| | S375D | | 5′-ccaagaagggccagtccgatcgccataaaaaaacaatggtc-3′ |
| | S373D+S37 | | 5′-ctgaagaccaggagggggccaggatactgaccgcgataaaaaaacaatggtc-3′ |
| | S389A | CKII | * |
| | S389D | | * |

*Already available.

promoter. Single-stranded DNA copies of the construct were harvested following R408 helper phage infection of transformed XL1-blue-MRF' $Kan^r$ cells. Two primers were annealed to the ssDNA, one to repair a mutated gene for ampicillin resistance and the primer coding for the required mutation in the murine *p53* gene. Annealed primer/templates were incubated for 3 h at 37°C with T4 DNA polymerase (7.5 units) and T4 DNA ligase (3 units) in a typical 30 μl reaction, transformed into XL1 blue-MRF' cells and selection carried out with Ampicillin at 125 μg $ml^{-1}$. Mutations in the *p53* gene of $Amp^r$ clones were confirmed initially by restriction digestion (where a new restriction site had been incorporated into the mutagenesis oligonucleotide) and by subsequent DNA sequencing.

Mutant *p53* genes were sub-cloned into $pGEM11ZF^-$ downstream of an SP6 promoter for expression in vitro. After restriction digestion, vector and insert bands were excised from a 0.8% agarose gel and isolated using a Geneclean kit (Bio101) or spin column kit (IGI) before ligation. DNA templates for in vitro transcription were prepared using the QIAGEN 'midiprep' protocol.

### In vitro transcription, translation and immunoprecipitation

In vitro transcription, translation and immunoprecipitation were as described previously (Milner et al, 1991) using plasmids encoding the wild-type or mutant *p53* gene under an SP6 promoter.

### Oligonucleotides for DNA binding

The following oligonucleotides were employed: p53 consensus (CON-DNA; 20 bp), 5′biotin GGACATGCCCGGGCATGTCC-3′ (Funk et al, 1992), lesion DNA (L-DNA; 60 bp) 5′ GCTCGAACC-CGTTCTCGGAGCACCCCTGCCCCAGCCCAACCGCTTTG-GCCGCCGCCCAGC-3′ (Lee et al, 1995) triple cytosine loops underlined. The oligonucleotides were annealed as follows: CON-DNA to itself, L-DNA to the biotinylated reverse sequence 5′biotin GCTGGGCGGCGGCCAAAGCGGTTCTGCAGTGCTCCGA-GAACGGGTTCGAGC-3′.

### DNA binding/proteolytic cleavage assays

Streptavidin-coated magnetic Dynabeads (Dynal) were used to harvest biotinylated DNA–protein complexes. Annealed CON-DNA or L-DNA oligonucleotides were bound to the beads (30 pmol of oligonucleotide + 15-μl beads) in TE pH 7.5; 1 M sodium chloride (NaCl) for 20 min at 20°C, washed with 2 × 400 μl TE–NaC1, and 2 × 400 μl DNA-binding buffer (20 mM Tris–HC1, pH 7.5; 100 mM NaCl; 0.1% NP40; 10% glycerol; 5 mM dithiothreitol). The beads suspended in DNA binding buffer were then incubated with $[^{35}S]$-methionine labelled p53 for 20 min at 20°C. The beads with DNA–p53 complexes were washed with 3 × 400 μl DNA-binding buffer and resuspended in 50 μl. Samples were incubated for 1 h at 37°C. The supernatant was removed and

© 1999 Cancer Research Campaign 

**Table 3** Summary of results

| p53 | Mutant | Conformation | | CON-DNA binding | L-DNA binding | Proteolytic Cleavage |
|---|---|---|---|---|---|---|
| | | 1620 | 240 | | | |
| Wild-type | | + | 0 | +++ | +++ | +++ |
| DNA contact mutants | R270H | + | 0 | 0 | +++ | +++ |
| | R245H | + | 0 | 0 | +++ | +++ |
| | K117A | + | 0 | + | +++ | +++ |
| Structural mutants | R172H | 0 | + | 0 | ++ | 0 |
| | A135V | 0 | + | 0 | ++ | 0 |
| | C173F | + | + | 0 | ++ | 0 |
| | C173M | + | + | 0 | ++ | 0 |
| | C173D | + | + | 0 | ++ | 0 |
| Phosphorylation mutants | S18A | + | 0 | +++ | +++ | +++ |
| | S18D | + | 0 | +++ | +++ | +++ |
| | S37D | + | 0 | +++ | +++ | +++ |
| | S312A | + | 0 | +++ | +++ | +++ |
| | S312D | + | 0 | +++ | +++ | +++ |
| | S373A | + | 0 | +++ | +++ | +++ |
| | S373D | + | 0 | +++ | +++ | +++ |
| | S375A | + | 0 | +++ | +++ | +++ |
| | S375D | + | 0 | +++ | +++ | +++ |
| | S373D+ S375D | + | 0 | +++ | +++ | +++ |
| | S389A | + | 0 | +++ | +++ | +++ |
| | S389D | + | 0 | +++ | +++ | +++ |

mixed with 30 µl Laemmli's buffer. The beads were washed with $3 \times 400$ µl DNA-binding buffer and resuspended in 30 µl Laemmli's buffer. Reaction products were analysed by sodium dodecyl sulphate polyacrylamide gel electrophoresis (SDS-PAGE) (15%) and autoradiography.

### Cell culture and immunoblotting

Clone 6 cells (rat embryo fibroblasts expressing the murine temperature sensitive mutant p53 A135V) (Michalovitz et al, 1990) were maintained in culture at 37°C in Iscove's medium supplemented with 10% fetal calf serum. For experimentation, the cells were sub-cultured and parallel cultures were incubated at either 32°C (p53 wild-type phenotype) or 37°C (p53 mutant phenotype) for 24 h prior to addition of *cis*-platinum (0, 1, 10 or 20 µg $ml^{-1}$). After a further 18 h the cells were trypsinized, washed twice with ice-cold phosphate-buffered saline, and counted prior to addition of Laemmli's buffer to give $10^4$ cells $\mu 1^{-1}$. Total cell lysates were analysed by SDS-PAGE and immunoblotting (as detailed in Okorokov et al, 1997) using anti-p53 monoclonal antibodies PAb248 (N-terminus), PAb240 (core domain) and PAb421 (C-terminus).

### RESULTS AND DISCUSSION

We were particularly interested to ask if naturally occurring mutants of p53 retain the ability to undergo proteolytic cleavage, and this series of mutants included: (i) DNA contact mutants, and (ii) structural mutants of murine p53 (Table 1). In particular this allowed us to ascertain whether murine p53 mutants, analogous to hotspot mutants commonly found in human cancer, abrogate the capacity of p53 to bind mismatched DNA and undergo proteolytic cleavage (the numbering of equivalent human mutants is included in Table 1). Analysis of structural mutants with abnormal conformation also enabled us to determine the importance of the tertiary structure of p53 for the cleavage reaction.

Each mutant was labelled with [$^{35}$S]-methionine during translation in rabbit reticulocyte lysate, and characterized by immunoprecipitation with a panel of monoclonal antibodies (Milner et al, 1991). Binding to a sequence-specific consensus DNA target (CON-DNA), (Funk et al, 1992) was also determined. Wild-type p53 was included as a positive control in every experiment: it displayed $246^+$ $1620^+$ $240^0$ immunoreactivity (Figure 1A) and showed sequence-specific DNA binding (Figure 1B), as predicted for the correctly folded, conformationally wild-type protein. No cleavage products were released from the p53–CON-DNA complexes (Figure 1B). Ability to undergo proteolytic cleavage was assayed by incubation with a mismatched DNA target which contained triple cytosine loops (L-DNA), (Lee et al, 1995; Molinari et al, 1996). On incubation with mismatched DNA, wild-type p53 was cleaved to yield the characteristic pattern of roughly equivalent levels of full length and 40-kDa protein bound to the DNA, with release of the 35-kDa cleavage product from the p53–DNA complexes (Figure 1B). These results are consistent with the previous observations of Molinari et al (1996) and Okorokov et al (1997).

The group of DNA contact mutants comprised R245H, R270H and K117A (equivalent to R248H, R273H and K120A in human p53, see Table 1). It is important to note that substitution of DNA contact residues on the surface of the p53 core domain is not predicted to perturb normal conformational folding and, as expected, the DNA contact mutants retained the wild-type immunoprecipitation phenotype (Figure 2A). Sequence-specific DNA binding by the DNA contact mutants was either completely lost (R245H, R270H) or greatly reduced (K117A) (Figure 2B). The apparent low level binding by K117A to CON-DNA may reflect interaction with the dsDNA ends of the oligonucleotide. Despite loss of sequence-specific DNA binding, the binding to

 *© 1999 Cancer Research Campaign*

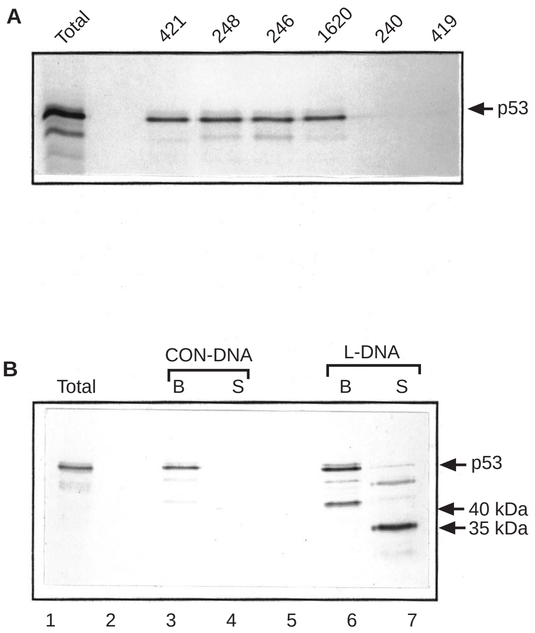

**Figure 1** **(A)** Immunoprecipitation of wild-type, in vitro translated murine p53 with a panel of monoclonal antibodies, as detailed by Milner et al (1991). The [$^{35}$S]-labelled protein displays a typical wild-type conformational phenotype, indicated by immunoreactivity with PAb246 and PAb1620, and lack of reactivity with PAb240. PAb421 and PAb248 detect carboxy and amino terminal epitopes respectively, and PAb419 is a negative control antibody. Proteins were resolved by 15% SDS–PAGE and visualized by autoradiography. **(B)** DNA binding and proteolytic cleavage of wild-type p53 (as detailed by Molinari et al (1996)). Briefly, biotinylated DNA was bound to streptavidin coated magnetic beads (Dynal) and the beads were washed to remove unbound oligonucleotides. In vitro translated [$^{35}$S]-labelled p53 was incubated with the DNA-coated beads and the beads were then washed to remove any unbound protein. The beads, with bound p53–DNA complexes, were resuspended in DNA-binding buffer (Molinari et al, 1996) and incubated at 37°C for 1 h. The incubated complexes were separated into beads (B) and supernatant (S) fractions, representing DNA bound and released fractions of the protein respectively. Equivalent aliquots of each sample were resolved by SDS-PAGE and visualized by autoradiography. An aliquot of the ‘total’ translation mix was used as a marker (lane 1). Assays were carried out with a sequence-specific target, CON-DNA (lanes 3 and 4) and with a mismatched DNA target, lesion DNA (L-DNA; lanes 6 and 7) as indicated

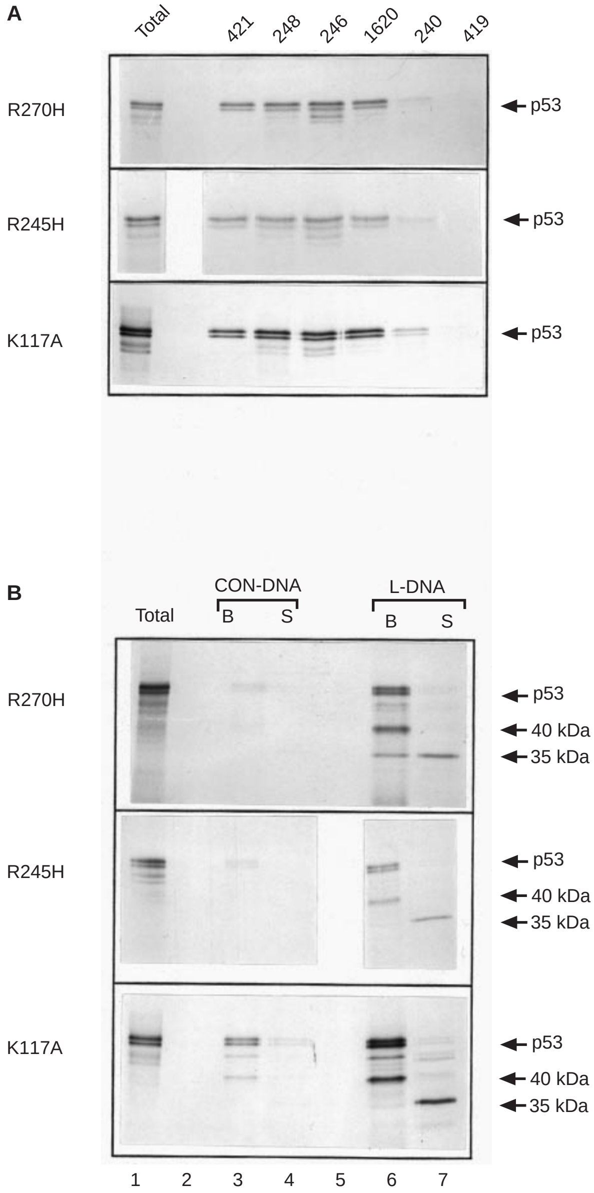

**Figure 2** **(A)** Immunoprecipitation profile and **(B)** DNA binding/proteolytic cleavage of murine *p53* DNA contact mutants (see Figure 1 legend for details). The Altered Sites™ In Vitro Mutagenesis System (Promega) was used to generate mutations in the wild-type murine *p53* gene mismatched DNA appeared unaffected by substitution of DNA contact residues R245, R270 and K117 since all three mutants bound the mismatched DNA target (Figure 2B). Moreover, binding to mismatched DNA was accompanied by proteolytic cleavage to generate the 40-kDa and 35-kDa cleavage products of the mutant protein (Figure 2B). Indeed, the ability of DNA contact mutants to bind the mismatched DNA target and undergo proteolytic cleavage was very similar to that of the wild-type protein (compare Figure 1B and Figure 2B).

Proteolytic cleavage of p53 may represent an autocatalytic process (Molinari et al, 1996; Okorokov et al, 1997). In which case it would be predicted to involve residues exposed on the native p53 protein. A number of naturally occurring serine catalytic dyads are known to exist on other proteins (reviewed in Paetzel and Dalbey, 1997), and computer-based analysis of the central core domain of p53 suggested that K117 may represent part of a catalytic dyad by association with S118 or C274. The positioning of these residues on the surface of p53 is indicated in Figure 3. In the light of this information we reasoned that K117 may play a catalytic role in the proteolytic cleavage of p53. However, we now show that substitution of K117 to alanine (which would be unable to associate with S118 or C274 in dyad formation) does not affect cleavage, indicating that K117 is dispensable for the proteolytic cleavage reaction of p53 and is therefore unlikely to participate in the catalytic process.

The second group of p53 mutants were structural (listed in Table 1). All showed loss of wild-type conformational structure, as indicated by exposure of the 240 epitope (normally cryptic for wild-type p53), (Gannon et al, 1990), loss of 1620 reactivity and loss of sequence-specific DNA binding (see Figure 4A for examples; summarized in Table 3). Despite loss of structural integrity and sequence-specific DNA binding ability (Figure 4B), these mutants retained the capacity to bind mismatched DNA (Figure 4B) indicating that altered conformational structure does not impede binding to a mismatched DNA target. However, there was no

*© 1999 Cancer Research Campaign*

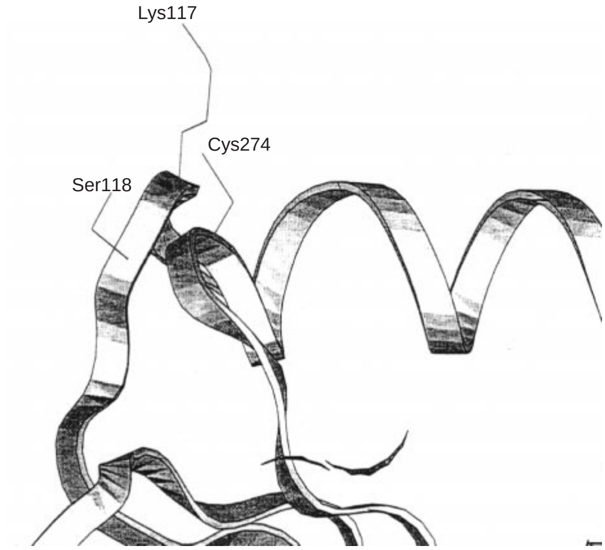

**Figure 3** Region on the surface of the core of p53 that represents a putative catalytic site for autoproteolytic cleavage. For the purposes of orientation, the carboxy terminal α-helix of the core crystal structure is on the right of the figure. Theoretically, K117 might associate with S118 or C274 to form a catalytic dyad. The human *p53* core crystal structure (Cho et al, 1994) was analysed using the program Molviewer (Protein Structure Group, Department of Chemistry, University of York). Numbering of residues according to murine *p53*

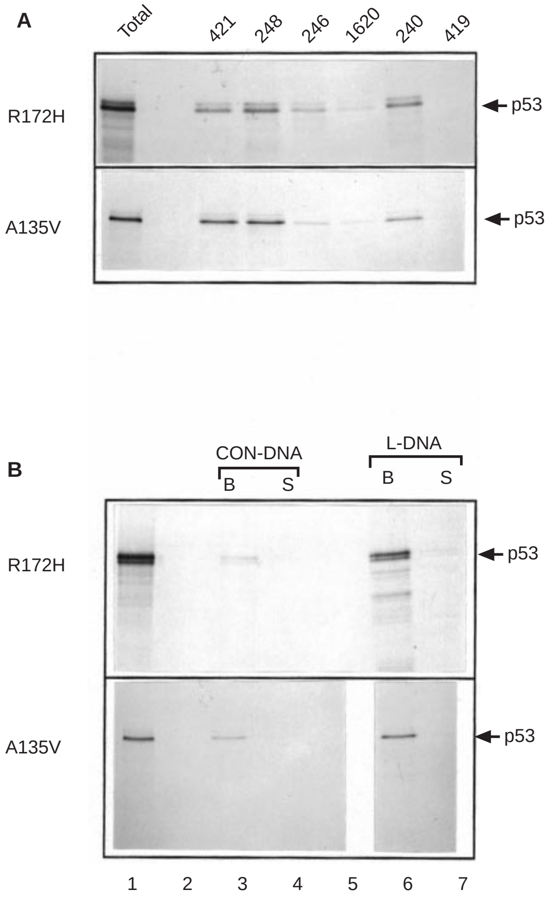

**Figure 4** **(A)** Immunoprecipitation profile and **(B)** DNA binding/proteolytic cleavage of murine *p53* structural mutants (see Figure 1 legend for details; see Figure 2 legend for details of mutagenesis)

evidence of proteolytic cleavage and no release of the 35-kDa product from the p53–DNA complexes (Figure 4B). We therefore conclude that wild-type tertiary structure is crucial for the generation and release of the 35-kDa cleavage product.

Further experiments using clone 6 rat embryo fibroblasts also indicate that cleavage products of p53 are generated in vivo following DNA damage and that the effect is conformation dependent. Clone 6 cells stably express a temperature-sensitive mutant of murine *p53*, A135V, and our in vitro results clearly demonstrate that p53 A135V binds damaged DNA but fails to undergo cleavage to generate either the 40-kDa or 35-kDa cleavage products (Figure 4B). Use of clone 6 cells allowed us to test the effect of p53 A135V conformation on the cleavage response to DNA damage in vivo since, at 37°C, the protein adopts the mutant phenotype, whilst at 32°C p53 A135V adopts the wild-type phenotype and results in cell growth arrest and apoptosis (Michalovitz et al, 1990; and results not shown). Evidence of p53 cleavage in response to cisplatin-induced DNA damage was observed in those cells cultured at 32°C (Figure 5). Exposure to cisplatin was for 18 h and the observed 40-kDa cleavage product of p53 was reactive with PAb240 and PAb421 but non-reactive with PAb248, indicating N-terminal cleavage of p53. This is consistent with previous results demonstrating a 40-kDa cleavage product of wild-type p53 in MCF7 cells following 18-h treatment with cisplatin (Molinari et al, 1996). The 35-kDa cleavage product is not expected in these samples since it appears only transiently, within 1 h exposure to cisplatin (Molinari et al, 1996). The full-length p53 band observed at 32°C (Figure 5) is predicted to represent both wild-type and mutant phenotypes of p53 A135V since complete conversion to the wild-type phenotype takes several days at 32°C (Michalovitz et al, 1990). In contrast to the cells cultured at 32°C there was no evidence of the 40 kDA product at 37°C (Figure 5), indicating that p53 A135V expressed in clone 6 cells only undergoes cleavage when it adopts the wild-type conformation. Thus the conformation of p53 appears critical for its cleavage in response to DNA damage both in vitro and in vivo.

The mechanisms for regulation of p53 function in vivo are not fully understood. One way in which p53 may be regulated is by phosphorylation. The amino and carboxy terminal domains of p53 can be phosphorylated by a number of kinases in vitro, some of which may regulate p53 function in vivo (reviewed by Meek, 1994; Steegenga et al, 1996). We next asked if phosphorylation might be a way of regulating the binding of p53 to damaged DNA and/or proteolytic cleavage induced by DNA damage. For this purpose, we analysed a series of 12 phosphorylation site mutants of murine p53 (listed in Table 2) and assayed for their capacity to bind mismatched DNA, and undergo proteolytic cleavage in vitro. Serine residues were mutated to alanine or aspartic acid, to simulate the dephosphorylated and phosphorylated residues respectively. The series of mutants included a double serine to aspartic acid mutant for S373 and S375 since these two residues have been implicated in phosphorylation by protein kinase C (PKC) (Takenaka et al, 1995; Milne et al, 1996). All the phosphorylation site mutants behaved like wild-type p53 in our assay system (results summarized in Table 3). This indicates that phosphorylation at single sites may not influence DNA binding and proteolytic cleavage. We cannot, however, rule out the possibility that multiple phosphorylation/dephosphorylation may regulate the cleavage reaction.

© 1999 Cancer Research Campaign

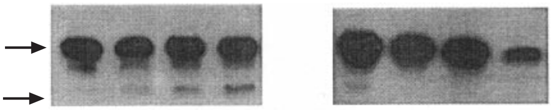

**Figure 5** Cleavage products of p53 generated in clone 6 cells in response to cisplatin treatment. Parallel cultures of clone 6 cells were cultured at 32°C or at 37°C for 24 h before treatment with cisplatin for a further 18 h. Total cell lysates were prepared (Materials and Methods) and analysed by immunoblotting; results shown employed PAb240 to detect *p53* and its cleavage products (Okorokov et al, 1997). Upper arrow indicates full-length p53 A135V which adopts the wild-type phenotype at 32°C and the mutant phenotype at 37°C (see text). Lower arrow indicates the position of the 40-kDa molecular weight marker. The doses of cisplatin (CP) used in a series of parallel cultures are as indicated. Note that reprobing the blots for actin showed equivalent loading for all lanes and the lower level of *p53* detected in 37°C cells treated with 20-µg cisplatin reflects a drug dosage affect under these conditions. The observed 40-kDa *p53* cleavage product generated in response to cisplatin-induced DNA damage of cells at 32°C is consistent with partial conversion of p53 A135V to the wild-type protein conformation after 24 h incubation at 32°C

Two major points emerge from the results of this study (summarized in Table 3). First, all p53 mutants analysed were able to bind to mismatched DNA. This suggests that in human cancer, mutant p53 retains the capacity to bind sites of DNA damage, even though it is deficient in activating a normal p53 response to the damage. Secondly, for proteolytic cleavage of the protein to take place, the central core domain must be folded into the wild-type conformation. The fact that loss of structural integrity correlates with loss of capacity for proteolytic cleavage is consistent with the idea that p53 cleavage is an autocatalytic process requiring the correct orientation of residues involved in the reaction. Where mutation gives rise to a misfolded protein, those residues that would normally interact to facilitate catalysis would be disrupted, thus preventing cleavage (as observed for structural mutants; Figure 4B and Table 3). Further studies are planned to determine which residues are involved in catalytic site formation for proteolytic cleavage of p53.

A third point concerns DNA contact mutants of p53 which occur with high frequency in human cancer. For example, mutations affecting R248 and R273 account for approximately 18% of p53 mutations in cancer. Such mutations cause loss of p53 transcriptional activation function, even though the protein retains normal structural conformation. We now demonstrate that DNA contact mutants retain the capacity to undergo proteolytic cleavage in response to mismatched DNA in vitro, with release of a 35-kDa cleavage product which is indistinguishable from that observed with the wild-type protein. The 35-kDa product released from wild-type p53 encompasses an intact core domain and is activated as an aminopeptidase (Okorokov et al, 1997). It may also be activated for other functions, such as 3′–5′ exonuclease activity (Mummenbrauer et al, 1996), and/or play a role in signal transduction cascades in the cell. In the later context it is relevant to note that the core domain of p53 has potential for interaction with target proteins such as 53BP2 via a novel SH3-binding motif (Thukral et al, 1994; Gorina and Pavletich 1996). Moreover, in-vivo expression of the 35-kDa core cleavage product induces G1 arrest when transfected into p53–/– murine embryo fibroblasts (Axe TJ et al, manuscript submitted). Since this cleavage product is released from full-length p53 R248H (Figure 2), these combined observations indicate the possibility of inducing growth suppression of tumour cells expressing p53 R248H and other DNA contact mutants.

### ACKNOWLEDGEMENTS

We are grateful to Pierre May for early discussions relating to this project. This work was funded by a programme grant from Yorkshire Cancer Research (to JM). We thank Meg Stark for photographic work.

### REFERENCES

Caelles C, Helmberg A and Karin M (1994) p53-dependent apoptosis in the absence of transcriptional activation of p53-target genes. *Nature* **370**: 220–223

Cho Y, Gorina S, Jeffrey PD and Pavletich NP (1994) Crystal structure of a p53 tumor suppressor–DNA complex: understanding tumorigenic mutations. *Science* **265**: 346–355

El-Diery WS, Tokino T, Velculescu VE, Levy DB, Parsons R, Trent JM, Lin D, Mercer WE, Kinzler KW and Vogelstein B (1993) WAF1, a potential mediator of p53 tumour suppression. *Cell* **75**: 817–825

Funk WD, Pak DJ, Karas RH, Wright WE and Shay JW (1992) A transcriptionally active DNA-binding site for human p53 protein complexes. *Mol Cell Biol* **12**: 2866–2871

Gannon JV, Greaves R, Iggo R and Lane DP (1990) Activating mutations in p53 produce a common conformational effect. A monoclonal antibody specific for the mutant form. *EMBO J* **9**: 1595–1602

Gorina S and Pavletich NP (1996) Structure of the p53 tumor suppressor bound to the ankyrin and SH3 domains of 53BP2. *Science* **274**: 1001–1005

Gottlieb TM and Oren M (1996) p53 in growth control and neoplasia. *Biochim Biophys Acta* **1287**: 77–102

Haupt Y and Oren M (1996) p53-mediated apoptosis: mechanisms and regulation. *Behring Inst Mitt* **97**: 32–59

Haupt Y, Rowan S, Shaulian E, Vousden KH and Oren M (1995) Induction of apoptosis in HeLa cells by transactivation-deficient p53. *Genes Dev* **9**: 2170–2183

Jayaraman J and Prives C (1995) Activation of p53 sequence-specific DNA binding by short single strands of DNA requires the p53 C-terminus. *Cell* **81**: 1021–1029

Ko LJ and Prives C (1996) p53: puzzle and paradigm. *Genes Dev* **10**: 1054–1072

Lee S, Elenbaas B, Levine A and Griffith J (1995) p53 and its 14 kDa C-terminal domain recognize primary DNA damage in the form of insertion/deletion mismatches. *Cell* **81**: 1013–1020

Levine AJ (1997) p53, the cellular gatekeeper for growth and division. *Cell* **88**: 323–331

Meek DW (1994) Post-translational modification of p53. *Semin Cancer Biol* **5**: 203–210

Michalovitz D, Halevy O and Oren M (1990) Conditional inhibition of transformation and of cell proliferation by a temperature-sensitive mutant of p53. *Cell* **62**: 671–680

Milne DM, McKendrick L, Jardine LJ, Deacon E, Lord JM and Meek DW (1996) Murine p53 is phosphorylated within the PAb421 epitope by protein kinase C in vitro, but not in vivo, even after stimulation with the phorbol ester o-tetradecanoylphorbol 13-acetate. *Oncogene* **13**: 205–211.

Milner J, Metcalf EA and Cook A (1991) Tumour suppressor p53: analysis of wild-type and mutant p53 complexes. *Mol Cell Biol* **11**: 12–19

Miyashita T and Reed JC (1995) Tumor suppressor p53 is a direct transcriptional activator of the human bax gene. *Cell* **80**: 293–299

Molinari M, Okorokov AL and Milner J (1996) Interaction with damaged DNA induces selective proteolytic cleavage of p53 to yield 40-kDa and 35-kDa fragments competent for sequence-specific DNA binding. *Oncogene* **13**: 2077–2086

Mummenbrauer T, Janus F, Muller B, Wiesmuller L, Deppert W and Grosse F (1996) p53 Protein exhibits 3′-to-5′ exonuclease activity. *Cell* **85**: 1089–1099

Okorokov AL, Ponchel F and Milner J (1997) Induced N- and C- terminal cleavage of p53: a core fragment of p53, generated by interaction with damaged DNA, promotes cleavage of the N-terminus of full-length p53, whereas ssDNA induces C-terminal cleavage of p53. *EMBO J* **16**: 6008–6017

Paetzel M and Dalbey RE (1997) Catalytic hydroxyl/amine dyads within serine proteases. *Trends Biochem Sci* **22**: 28–31

Steegenga WT, van der Eb AJ and Jochemsen AG (1996) How phosphorylation regulates the activity of p53. *J Mol Biol* **263**: 103–113

© 1999 Cancer Research Campaign 

Takenaka I, Morin F, Seizinger BR and Kley N (1995) Regulation of the sequence-specific DNA binding function of p53 by protein kinase C and protein phosphatases. *J Biol Chem* **270**: 5405–5411

Thukral SK, Blain GC, Chang KK, and Fields S (1994) Distinct residues of human p53 implicated in binding to DNA, simian virus 40 large T antigen, 53BP1, and 53BP2. *Mol Cell Biol* **14**: 8315–8321

*© 1999 Cancer Research Campaign*
